# Putrescine mitigates intestinal atrophy through suppressing inflammatory response in weanling piglets

**DOI:** 10.1186/s40104-019-0379-9

**Published:** 2019-09-10

**Authors:** Bangmin Liu, Xianren Jiang, Long Cai, Xuemei Zhao, Zhaolai Dai, Guoyao Wu, Xilong Li

**Affiliations:** 1 Key Laboratory of Feed Biotechnology of the Ministry of Agriculture and Rural Affairs, Feed Research Institute, Chinese Academy of Agricultural Sciences, No. 12 Zhongguancun South St., Haidian district, Beijing, 100081 China; 2 College of Animal Science and Technology, China Agricultural University, Beijing, 100193 China; 3 Departments of Animal Science and of Medical Physiology, Texas A&M University, College Station, TX 77843 USA

**Keywords:** Cell migration, Cell proliferation, Intestinal atrophy, Mucosal immune response, Putrescine, Weaning stress

## Abstract

**Background:**

Polyamines are essential for cell growth and beneficial for intestinal maturation. To evaluate the effects of putrescine on alleviating intestinal atrophy and underlying molecular mechanisms, both *in vivo* feeding trial and *in vitro* cell culture were conducted. Weanling pigs were fed a diet supplemented with 0, 0.1%, 0.2% or 0.3% putrescine dihydrochloride, whereas porcine intestinal epithelial cells (IPEC-J2) were challenged with lipopolysaccharide (LPS) in the presence of 200 μmol/L putrescine.

**Results:**

Dietary supplementation with 0.2% putrescine dihydrochloride decreased the incidence of diarrhea with an improvement in intestinal integrity. Inhibition of ornithine decarboxylase activity decreased the proliferation and migration of IPEC-J2 cells, and this effect was alleviated by the supplementation with putrescine. The phosphorylation of extracellular signal regulated kinase and focal adhesion kinase was enhanced by putrescine. LPS increased the expression of inflammatory cytokines [tumor necrosis factor α (TNF-α), interleukin 6 (IL-6) and IL-8], and inhibited cell proliferation and migration in IPEC-J2 cells. Adding exogenous putrescine suppressed the expression of TNF-α, IL-6 and IL-8, and recovered cell migration and proliferation in LPS-treated IPEC-J2 cells. Dietary putrescine supplementation also reduced the mRNA levels of TNF-α, IL-6 and IL-8 and their upstream regulator nuclear receptor kappa B p65 subunit in the jejunal mucosa of piglets.

**Conclusions:**

Dietary supplementation with putrescine mitigated mucosal atrophy in weanling piglets through improving anti-inflammatory function and suppressing inflammatory response. Our results have important implications for nutritional management of intestinal integrity and health in weanling piglets and other neonates.

**Electronic supplementary material:**

The online version of this article (10.1186/s40104-019-0379-9) contains supplementary material, which is available to authorized users.

## Introduction

The small intestine is the main site for the terminal digestion and absorption of nutrients, and is also the first barrier to prevent the invasion of diet-derived pathogens and other harmful substances. Intestinal integrity is prerequisite for normal function, and is essential to survival for animals and humans. Weaning is a stressful condition for neonates, both physically and psychologically. Due to the dramatic change of diet from highly digestible liquid milk to a plant-based solid diet, the structure and function of the small intestine is damaged in piglets, as characterized by villous atrophy, crypt hyperplasia and digestive dysfunction [1]. As a result, diarrhea and growth retardation occur during weaning. Repairing this mucosal damage needs knowledge about mechanisms for wound healing, which starts with early restitution (a process depending on the migration of the intestinal epithelial cells) and the subsequent healing (which replaces lost cells through proliferation of crypt cells) [2]. These events indicate that the proliferation and migration of epithelial cells play important roles in repairing mucosal damage and sustaining intestinal integrity.

Polyamines, include putrescine, spermidine and spermine, are positively charged aliphatic amines with a low molecular weight in all eukaryotic cells [3]. Putrescine, the precursor for spermidine and spermine, is produced from ornithine through ornithine decarboxylase (ODC), which is the rate-limiting step in polyamine biosynthesis. In humans, food is the major source of polyamines in the lumen with a majority of ingested polyamines being absorbed in the small intestine, while the gut microbiota is mainly responsible for the level of polyamines in the large intestine [4]. Polyamines have many intracellular bio-functions such as modulating DNA structure, mRNA translation and protein activity, as well as playing essential roles in promoting cell proliferation and migration [3, 5]. Because cell proliferation and migration are important in mucosal morphology and function, polyamines are required for repairing intestinal damage caused by weaning. Intestinal ODC is induced by early weaning in piglets [6], further indicating a possible role of polyamines in intestinal development. There are reports that oral administration of polyamines to sucking piglets may improve the gut morphology [7–9]. However, little is known about the underlying mechanisms. Intestinal integrity may be compromised by local inflammatory responses in the small intestine of weanling mammals [10], and emerging evidences suggests that polyamines may play roles in regulating the inflammatory response [11–13]. We are not aware of any studies investigating the effects of polyamines on the inflammatory response in enterocytes.

In view of the foregoing, the objective of this study was to evaluate the effects of dietary supplementation with putrescine on the recovery of the intestinal damage caused by weaning stress and to explore the underlying mechanism. Our hypothesis was that supplementation with putrescine supports mucosal integrity in weanling piglets through alleviating the epithelial atrophy caused by mucosal inflammation. Both *in vivo* feeding trial and *in vitro* cell culture with an inflammation model were employed to test this hypothesis.

## Methods

### Animals and experimental design

The animal study was approved by the Animal Care and Use Committee of the Feed Research Institute of the Chinese Academy of Agricultural Sciences. A total of 72 crossbred (Duroc × Landrace × Yorkshire) barrows (7.38 ± 0.15 kg) were weaned at 23 days of age and assigned randomly to 1 of 4 treatments according to body weight. Dietary treatments included a corn- and soybean-based diet supplemented with 0% (control group), 0.1%, 0.2% or 0.3% putrescine dihydrochloride (purity≥98%, Cat. # S30044–500 g, Shanghai Yuanye Biotechnology Co., Ltd., Shanghai, China). The levels of putrescine were chosen based on 2 times of the dosage administered orally to suckling piglets (5 mg/kg BW) [9]. There were 6 pens per treatment with 3 piglets per pen. Each pen had a slatted floor and a size of 2 m × 2 m. During the experiment, piglets had free access to drinking water and feed. Ventilation was achieved by using speed-controlled fans. The room temperature was initially set at 28 °C and decreased by 1 °C per week. Each pen was equipped with two water nipples and feed-trough. The diet for the piglets was prepared according to National Research Council (2012) nutrient requirements [14], and nutrient levels of the basal diet is shown in Table 1.

**Table 1:** Ingredient and nutrient composition of the basal diet (on an as-fed basis)

Item	g/kg
Ingredients	
Corn	223.7
Extruded corn	235.8
Soybean meal	150.0
Extruded soybean	145.0
Whey	150.0
Fish meal	55.0
Soybean oil	5.0
Dicalcium phosphate	5.5
Limestone (CaCO_3_)	6.5
Salt	2.5
Choline chloride (60%)	0.8
*L*-Lysine HCl	5.5
*DL*-Methionine	0.9
Threonine	0.8
Vitamin and mineral premix^1^	10.0
Zinc oxide	3.0
Analyzed nutrient content	
Crude protein	189.1
Calcium	8.5
Phosphorus	6.8
Free putrescine, mg/kg	10.3
Calculated nutrient content	
ME, MJ/kg	14.23
Lysine	13.0
Methionine	4.0
Threonine	7.3
Tryptophan	2.0

^1^ Premix supplied per kg of diet: vitamin A, 35.2 mg; vitamin D_3_, 7.68 mg; vitamin E, 128 mg; vitamin K_3_, 8.16 mg; vitamin B_1_, 4 mg; vitamin B_2_, 12 mg; vitamin B_6_, 8.32 mg; vitamin B_12_, 4.8 mg; niacin, 38.4 mg; calcium pantothenate, 25 mg; folic acid, 1.68 mg; biotin, 0.16 mg; iron (FeSO_4_·H_2_O), 171 mg; manganese (MnSO_4_·H_2_O), 42.31 mg; copper (CuSO_4_·5H_2_O), 125 mg; selenium (Na_2_SeO_3_), 0.19 mg; cobalt (CoCl_2_), 0.19 mg; iodine [Ca(IO_3_)_2_], 0.54 mg

Due to the low feed intake of piglets within the first 5 days post-weaning [15], putrescine was administered orally into piglets to ensure putrescine intake by piglets. The amount of supplemental putrescine (0, 0.1%, 0.2% or 0.3% putrescine dihydrochloride in the diet) was calculated based on a daily feed intake of 200 g for each piglet. Putrescine dihydrochloride was dissolved in saline before its oral administration to piglets via a 10-mL dispensable syringe.

At the end of a 14-day trial, pigs were weighed and total feed consumption during the whole trial was measured for each pen. The average daily gain (ADG), average daily feed intake (ADFI), and gain to feed ratio (G:F) were calculated. The incidence of diarrhea was recorded for each piglet per day.

### Sample collection

At the end of the animal trial, one piglet was randomly selected from each pen for euthanasia by intravenous injection of pentobarbital sodium (6 mg/kg BW). The body weight of selected piglets was listed in Additional file 1: Table S1. A length of approximate 15 cm of the jejunum beginning 50 cm distal to the pylorus of each piglet was removed. Its first 3 cm segment was fixed in fresh 4% paraformaldehyde for 24 h and stored in 70% ethanol. The remaining segment was cut longitudinally to expose mucosa and washed 3 times in phosphate-buffered saline (PBS) to remove the mucus and digesta. Then, the mucosa was gently scraped off with a glass microscope slide and placed in a sterile cryogenic vial (Corning Inc., NY, USA), which was rapidly frozen in liquid nitrogen and then stored at − 80 °C until use for RNA and protein determination.

### Jejunum morphology

The specimens of jejunum were dehydrated in graded ethanol series and cleared with xylene and embedded in paraffin [1]. Then three pieces of 5 μm thick sections were prepared from the embedded samples, which were stained with hematoxylin-eosin. Under an optical microscope (Olympus CX31, Olympus Corporation, Tokyo, Japan), 10 fields were randomly selected to measure the villus height and the crypt depth, and calculate villus height: crypt depth (V:C) ratios.

### Cell culture

The porcine intestinal epithelial cell (IPEC-J2) (from Dr. Guoyao Wu’s laboratory at Texas A&M University), a well-established porcine non-transformed intestinal cell line developed from mid-jejunum of newborn piglet [16], was cultured in Dulbeco’s modified Eagle’s medium/F12 (DMEM/F12, Thermo Fisher Scientific, MA, USA) supplemented with 1% pen-strep (Thermo Fisher Scientific, MA, USA), 0.1% ITS (5 μg/L insulin, 5 μg/L transferrin and 5 ng/L selenious acid, Corning Inc., NY, USA), 0.01% epidermal growth factor (5 μg/L, Corning Inc., NY, USA) and 5% fetal bovine serum (FBS) (ThermoFisher, MA, USA) at 37 °C in an incubator with the humidified atmosphere composed of 95% air and 5% CO_2_. We used passage 13–15 cells in these experiments.

### Cell proliferation assay

IPEC-J2 cells were seeded at 0.4 × 10^5^ cells/mL (0.4 mL per well) in 24 well Costar plates (Corning, New York, USA) with 4 replications (wells) per treatment. When the cells were attached to the plate bottom (~ 4 h), culture media were removed and cells were washed with PBS 3 times, followed by the addition of 0.4 mL culture medium (containing 2% FBS) per well for each treatment. In each well, the concentration of putrescine (putrescine dihydrochloride, Sigma-Aldrich, Co., MO, USA) was 0, 10, 25, 50, 100 or 200 μmol/L, respectively. In some experiments, 5 mmol/L difluoromethyl ornithine (DFMO, MedChemExpression, NJ, USA), a specific inhibitor of ODC, was used to suppress the production of putrescine by the cells. Culture media were changed every 24 h. The cell counting kit (CCK-8, MedChemExpression, NJ, USA) was used to determine the growth of cells according to the manufacturer’s protocol. Briefly, 40 μL CCK-8 was added to each well, after incubation for 3 h, the optical density (OD) values were measured at 450 nm with an Epoch Microplate Spectrophotometer (BioTek Instruments, Inc., VT, USA). Cell numbers were calculated from OD values according to the formula developed by a standard curve.

### Cell migration assay

Two milliliters of cell suspension (0.4 × 10^5^ cells/mL) were seeded into 6 well Costar plates (Corning, New York, USA) for 24 h. The cells were cultured for 48 h in different treatments (with or without 200 μmol/L putrescine or DFMO). Mitomycin C (2 μg/mL, MedChemExpression, NJ, USA) was added 24 h before scratching to fully inhibit cell proliferation [17]. A straight scratch was made by using a 200-μL pipette tip. The cells were washed twice with PBS, and the images were acquired at 0 h and 8 h post scratching at the same location by an inverted microscopy (Axio Vert.A1, Zeiss, Germany). The area of cell migration was measured and expressed as the surface area covered by cells (in percentage).

### Establishment of *in vitro* cell inflammation model

IPEC-J2 cells were challenged with 100 μg/mL lipopolysaccharides (LPS, *E. coli* O55:B5, Sigma-Aldrich, Co., MO, USA) to establish an *in vitro* cell inflammation model. For measuring the effect of LPS challenge on cell proliferation, IPEC-J2 cells were seeded in 96 well plates, and pretreated for 48 h with or without 200 μmol/L putrescine. The cells were challenged with or without 100 μg/mL LPS for 4 h, and cell proliferation rate was measured with EdU kit (Beyotime technology, Shanghai, China) according to the protocol. Briefly, cells were labeled with EdU, and fixed for 15 min at 25 °C, followed by washing. Cells were incubated with 0.3% H_2_O_2_, followed by washing, addition of 50 μL reaction buffer to each well, and incubation for 30 min in dark at 25 °C. Streptavidin-HRP was used for labeling, and color was developed after the addition of the TMB substrate. The OD values were measured at 620 nm with an Epoch Microplate Spectrophotometer (BioTek Instruments, Inc., VT, USA). For measuring the effect of LPS challenge on cell migration, IPEC-J2 cells were seeded in 6 well plates, and pretreated for 48 h with or without 200 μmol/L putrescine, followed by the addition of 2 μg/mL Mitomycin C for 24 h. The cells were then challenged with or without 100 μg/mL LPS for 4 h before scratching. Images were taken at 0 h and 8 h post scratching to calculate an area covered by cell migration.

### Extraction and determination of polyamines in diet, culture media and cells

Free polyamines in the diet were extracted according to the previous method with some modification [18]. Briefly, 0.5 g diet was weighed and homogenized in 3 mL of 5% ice-cold perchloric acid (*v*/*v*), followed by incubation for 30 min on ice. After centrifugation at 12,000×*g* for 30 min at 4 °C, 100 μL of the supernatant fluid was mixed with an equal volume of 1.5 mol/L HClO_4_ and then neutralized by addition of 100 μL of 2 mol/L of K_2_CO_3_. The tubes were centrifuged at 13,000×*g* for 5 min, and the resulting supernatant fluid was used for HPLC determination of free polyamines. For determination of polyamines content in culture media and cells, IPEC-J2 cells were harvested by centrifugation at 1,000×*g* for 10 min. The supernatant fluid was obtained and deproteinized as described previously, whereas the cells were washed twice with ice-cold PBS (pH 7.4). Cells were lysed in 100 μL ice-cold PBS (pH 7.4) by sonication, and deproteinized as described previously. Polyamines in samples were determined by HPLC (Alliance e2695 HPLC system, Waters Corporation, MA, USA) using the OPA-NAC method [19].

### Quantitative real-time PCR (qPCR)

Total RNA was extracted from the piglet jejunal mucosa and IPEC-J2 cells with the use of the Trizol reagent (Thermo Fisher Scientific, MA, USA) according to the manufacturer’s instructions. Total RNA was quantified by Epoch Microplate Spectrophotometer (BioTek Instruments, Inc., VT, USA). Total RNA (1 μg) was used to generate cDNA by the first-strand synthesis kit (Thermo Fisher Scientific, MA, USA). qPCR was performed using SYBR Green (Thermo Fisher Scientific, MA, USA) according to the manufacturer’s instructions on an ABI 6 flex real-time PCR instrument (Thermo Fisher Scientific, MA, USA). The primers for qPCR were listed in Additional file 2: Table S2. The comparative CT method was used to determine fold changes in gene expression, calculated as 2^-ΔΔCT^. The relative expression of each target gene was normalized to the mRNA level of the *GAPDH* gene.

### Western blotting

Cells were washed 3 times with cold PBS and lysed for total protein extraction in the radio-immunoprecipitation assay buffer (Thermo Fisher Scientific, MA, USA) with 1% protease inhibitors and a phosphatase inhibitor cocktail (Thermo Fisher Scientific, MA, USA) for 30 min at 4 °C. The cell lysate was centrifuged at 12,000×*g* for 10 min and the supernatant fluid was analyzed for protein with the bicinchoninic acid (BCA) kit (Applygen, Beijing, China). Equivalent amounts of protein (25 μg) with 4× loading buffer (Bio-Rad Laboratories Inc., CA, USA) were denatured in boiling water for 10 min, followed by cooling on ice. Denatured proteins were separated by SDS-PAGE (12% gel) and transferred into the polyvinylidene difluoride membrane (Bio-Rad Laboratories Inc., CA, USA) for 2 h at 200 mA using the Bio-Rad Mini-PROTEAN Tetra electrophoresis system (Bio-Rad Laboratories Inc., CA, USA). Membranes were blocked for 1 h in 5% bovine serum albumin in Tris-buffered saline with Tween 20 (TBST) buffer, and then incubated with a primary antibody at 4 °C overnight with gentle rocking. All antibodies used in this research are listed in Additional file 3: Table S3. Antibodies were validated with porcine intestinal cells and mucosa before use for this study (see additional file 1 for “Validation of antibodies”). Prestained protein standards (Bio-Rad Laboratories Inc., CA, USA, Cat. #1610375) and unstained protein standards (Bio-Rad Laboratories Inc., CA, USA, Cat. #1610363) were used as molecular weight ladders. GAPDH was the house-keeping protein, and run on the same gel as the target proteins. After being washed three times with TBST, the membranes were incubated at 25 °C for 1 h with a secondary antibody, washed with TBST, and developed with enhanced chemiluminescence prime reagents (Bio-Rad Laboratories Inc., CA, USA). The images were detected by the ChemiDoc MP Imaging System (Bio-Rad Laboratories, Inc., CA, USA).

### Statistical analysis

The Chi-square test was used for the analysis of diarrhea incidence. Data were analyzed as a completely randomized block design, using the GLM procedure of SAS v. 9.2 (SAS Institute Inc., NC, USA). The model included the treatment effect, and pen was the experimental unit for growth performance, while individual piglet was the experimental unit for intestinal morphology. Differences among treatment means were determined by the Duncan’s multiple comparison test. Differences were considered statistically significant at *P* ≤ 0.05.

## Results

### Growth performance, diarrhea incidence and small-intestinal morphology

During a 2-week animal trial, no differences were found for ADG, ADFI and G:F among different groups. However, piglets in the 0.2% putrescine dihydrochloride group had a lower incidence of diarrhea, compared with the control group during day 0–7 and day 0–13 post-weaning (*P* < 0.05), and tended to have a decreased incidence of diarrhea during day 8–13 post-weaning (*P* = 0.06) (Table 2). There was no difference in villus height among groups, but the crypt depth was decreased in the 0.1% and 0.2% putrescine dihydrochloride groups, compared with the control group (*P* < 0.05). In comparison with the control group, dietary supplementation with 0.1% to 0.3% putrescine dihydrochloride enhanced the villus height to crypt depth ratio (*P* < 0.05) (Table 3). Dietary supplementation with 0.2% putrescine dihydrochloride increased the mRNA levels of zona occludens 1 (ZO-1) and occludin, and tended to enhance the mRNA level of claudin-1 (Additional file 4: Figure S1).

**Table 2:** Effects of dietary putrescine supplementation on growth performance and diarrhea incidence in weanling piglets

Item	Putrescine dihydrochloride, %				SEM	*P*-value
	0	0.1	0.2	0.3		
Body weight, kg						
D 0	7.38	7.38	7.38	7.37	0.55	0.93
D 14	9.54	9.40	9.84	9.23	0.70	0.59
ADG, g/d	155	127	176	125	19	0.19
ADFI, g/d	290	230	307	242	23	0.12
G:F	0.54	0.53	0.57	0.53	0.05	0.92
Diarrhea incidence, %						
D 0–7	16.67^a^	14.69^ab^	7.64^b^	19.44^a^	–	0.03
D 8–13	24.07	18.63	10.19	17.59	–	0.06
D 0–13	19.84^a^	16.33^a^	8.73^b^	18.65^a^	–	< 0.01

Note: *n* = 6. *ADFI* average daily feed intake, *ADG* average daily gain, *G:F* gain to feed ratio *D* day

^a,b^Within a row, means not sharing the same superscript differ (*P* < 0.05)

**Table 3:** Effects of dietary supplementation with putrescine on jejunal morphology in weanling piglets

Item	Putrescine dihydrochloride, %				SEM	*P*-value
	0	0.1	0.2	0.3		
Villus height, μm	365	353	384	401	7.0	0.73
Crypt depth, μm	246^a^	208^b^	165^c^	228^a^	5.7	< 0.01
Villus height: Crypt depth	1.48^c^	1.70^b^	2.35^a^	1.76^b^	0.06	< 0.01

Note: *n* = 6

^a-c^Within a row, means not sharing the same superscript differ (*P* < 0.05)

### Effects of exogenous putrescine on the proliferation and migration of IPEC-J2 cell

**Fig. 1:**
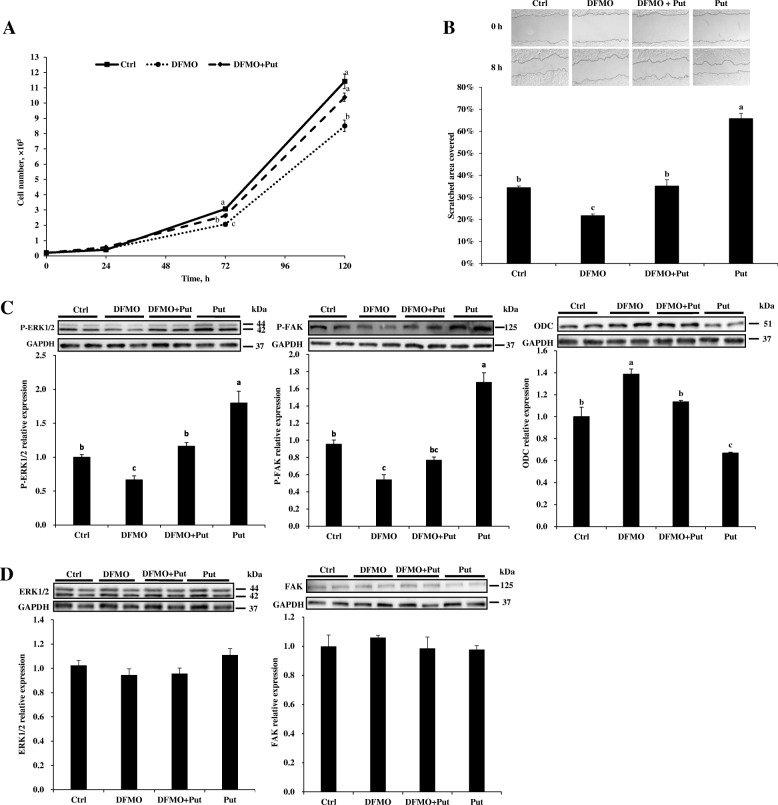
Effects of putrescine on cell proliferation and migration in IPEC-J2 cells. **a** Addition of putrescine to culture medium rescued the growth of cells treated with DFMO. The IPEC-J2 cells were cultured in 24-well plates with or without DFMO or putrescine, and cell numbers were determined at 0 h, 24 h, 72 h, and 120 h with the CCK-8 kit. **b** Pretreatment with putrescine prevented an inhibitory effect of DFMO on cell migration. IPEC-J2 were cultured in a 6-well plate with or without DFMO or putrescine. Cells were treated with mitomycin C for 24 h before scratching. Images were taken immediately after scratching (0 h) and at 8 h post scratching to calculate area covered by cell migration. The scratched borders were enhanced with black lines. **c** Western blotting of Phospho-ERK1/2, phospho-FAK and ODC. **d** Western blotting of total-ERK1/2 and total-FAK. Values are means ± SE, *n* = 4. Means with different letters are different (*P* < 0.05). CCK-8, cell counting kit-8; Ctrl, control; DFMO, difluoromethyl ornithine; IPEC-J2, porcine intestinal epithelial cells; P-ERK1/2, phospho-ERK1/2; P-FAK, phospho-FAK; Put, putrescine

Compared with the control, the addition of 12.5 μmol/L putrescine to culture medium increased cell numbers only at 120 h of culture; 25 μmol/L and 50 μmol/L putrescine supplementation increased cell numbers at 96 h and 120 h; however, 100 μmol/L and 200 μmol/L putrescine supplementation increased cell numbers at earlier times (24 h and 72 h) as well as at 96 h and 120 h (*P* < 0.05) (Additional file 5: Figure S2). Of those doses, 200 μmol/L putrescine had the greatest effect on stimulating cell proliferation; therefore, this dose was used for the subsequent experiments. DFMO inhibited cell proliferation at 72 h and 120 h. However, adding simultaneously exogenous putrescine partially rescued cell growth at 72 h, and completely recovered cell growth at 120 h (*P* < 0.05) (Fig. 1a).

Adding 2 μg/mL mitomycin C for 24 h fully inhibited IPEC-J2 cell proliferation (Additional file 6: Figure S3). The addition of 200 μmol/L putrescine increased cell immigration at 8 h after scratching (*P* < 0.05) (Fig. 1b). DFMO inhibited cell migration, but this inhibition was prevented by pretreatment with exogenous putrescine (Fig. 1b).

### Effects of exogenous putrescine on the levels of polyamines in IPEC-J2 cell

Addition of 200 μmol/L putrescine to culture medium increased putrescine concentration in cells (*P* < 0.05) (Table 4). The levels of spermine in both medium and cells were enhanced by 200 μmol/L putrescine. Adding 200 μmol/L putrescine slightly decreased spermidine concentration in medium, but had no effect on spermidine concentration in the cells.

**Table 4:** The concentrations of polyamines in culture medium and cells treated with putrescine or DFMO

Item	Ctrl	DFMO	DFMO+Put	Put	SEM	*P*-value
Medium polyamines, nmol/mL						
Putrescine	0.26^c^	0.15^c^	136.67^a^	111.33^b^	18.87	< 0.01
Spermidine	3.84^a^	1.82^b^	1.79^b^	2.33^b^	0.29	< 0.01
Spermine	29.33^c^	37.33^b^	51.67^a^	50.00^a^	2.86	< 0.01
Cell lysates polyamines, nmol/mL						
Putrescine	1.87^b^	0.66^c^	2.61^a^	2.54^a^	0.25	< 0.01
Spermidine	12.21^a^	4.46^b^	11.31^a^	11.35^a^	0.96	< 0.01
Spermine	10.78^b^	11.38^b^	16.09^a^	14.88^a^	0.70	< 0.01

Note: *n* = 4. *Ctrl* control, *DFMO* 5 mmol/L difluoromethyl ornithine, *Pu*, 200 μmol/L putrescine;

^a-c^ Within a row, means not sharing the same superscript differ (*P* < 0.05)

DFMO significantly decreased putrescine and spermidine concentrations in both medium and cells, but spermine concentrations did not change. Adding extracellular putrescine elevated the concentrations of putrescine, spermidine and spermine in DFMO-treated cells (*P* < 0.05).

### Putrescine activated ERK1/2 and FAK in IPEC-J2 cells and stimulated their proliferation and migration

Compared with the control, addition of putrescine to culture medium increased the phosphorylation of ERK1/2 and FAK (*P* < 0.05) (Fig. 1c). Phosphorylation of ERK1/2 and FAK was reduced by DFMO, but recovered by the simultaneous addition of putrescine (Fig. 1c). However, adding putrescine or DFMO did not alter the abundances of ERK1/2 and FAK in cells (Fig. 1d). Adding extracellular putrescine decreased the abundance of ODC in cells, and the opposite result was obtained in the presence of DFMO (*P* < 0.05) (Fig. 1c).

Ulixertinib (an ERK inhibitor) reduced cell growth (*P* < 0.05). Unlike DFMO, addition of putrescine did not rescue cell proliferation in the presence of ulixertinib (Additional file 7: Figure S4A). Ulixertinib also inhibited phosphorylation of ERK1/2, and this inhibition was not attenuated by the simultaneous addition of putrescine (*P* < 0.05) (Additional file 7: Figure S4B). Cell migration as well as phosphorylation of FAK was inhibited (*P* < 0.05) by defactinib (a FAK inhibitor), but pretreatment with putrescine did not rescue cell migration and FAK phosphorylation in the presence of defactinib (Additional file 8: Figure S5A, B). Pretreatment with ulixertinib or defactinib did not alter the abundances of ERK1/2 and FAK in cells (Additional file 8: Figure S4C, 5C).

### Putrescine alleviated the stimulatory effect of LPS on expression of TNF-α, IL-6 and IL-8 in IPEC-J2 cells

**Fig. 2:**
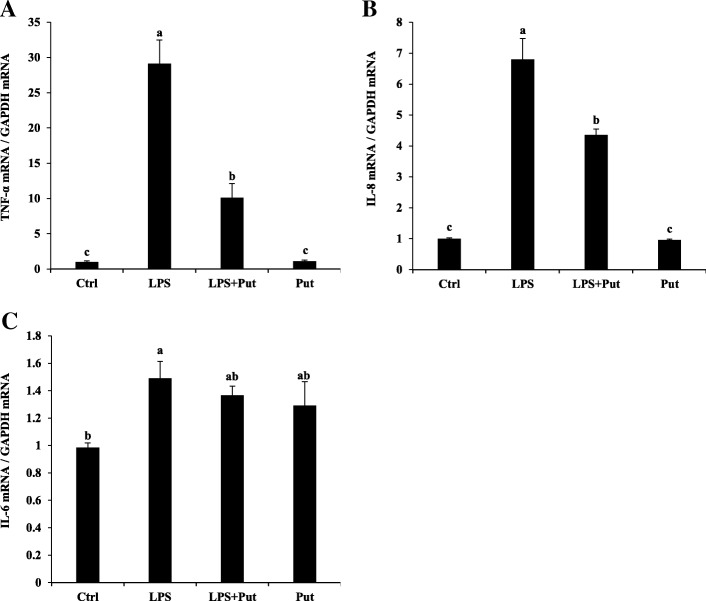
Putrescine attenuated gene expression of proinflammatory cytokines in LPS-treated IPEC-J2 cells. The IPEC-J2 cells were seeded in 6 well plates, and pretreated for 48 h with or without 200 μmol/L putrescine. The cells were challenged with or without 100 μg/mL LPS, and mRNA levels for proinflammatory cytokine TNF-α (**a**), IL-8 (**b**), and IL-6 (**c**) were quantified by qPCR at 4 h after challenge. Values are means ± SE, *n* = 4. Means with different letters are different (*P* < 0.05). Ctrl, control; GAPDH, glyceraldehyde-3-phosphate dehydrogenase; IL-6, interleukin 6; IL-8, interleukin 8; IPEC-J2, porcine intestinal epithelial cells; LPS, lipopolysaccharides; Put, putrescine; TNF-α, tumor necrosis factor α

Addition of 100 μg/mL LPS to culture medium dramatically enhanced mRNA levels of inflammatory cytokine TNF-α and chemokine IL-8 (Fig. 2a, b), with a slightly but significantly increase in mRNA levels of IL-6 (*P* < 0.05) (Fig. 2c). However, the elevated mRNA levels of TNF-α, IL-6 and IL-8 in the presence of LPS were attenuated by pretreatment with putrescine (*P* < 0.05) (Fig. 2a, b, c). Adding putrescine to culture medium in the absence of LPS had no effect on the mRNA levels of TNF-α and IL-8 (Fig. 2a, b), but tended to increase the mRNA levels of IL-6 (Fig. 2c).

### Putrescine rescued IPEC-J2 cell proliferation and migration in the presence of LPS

**Fig. 3:**
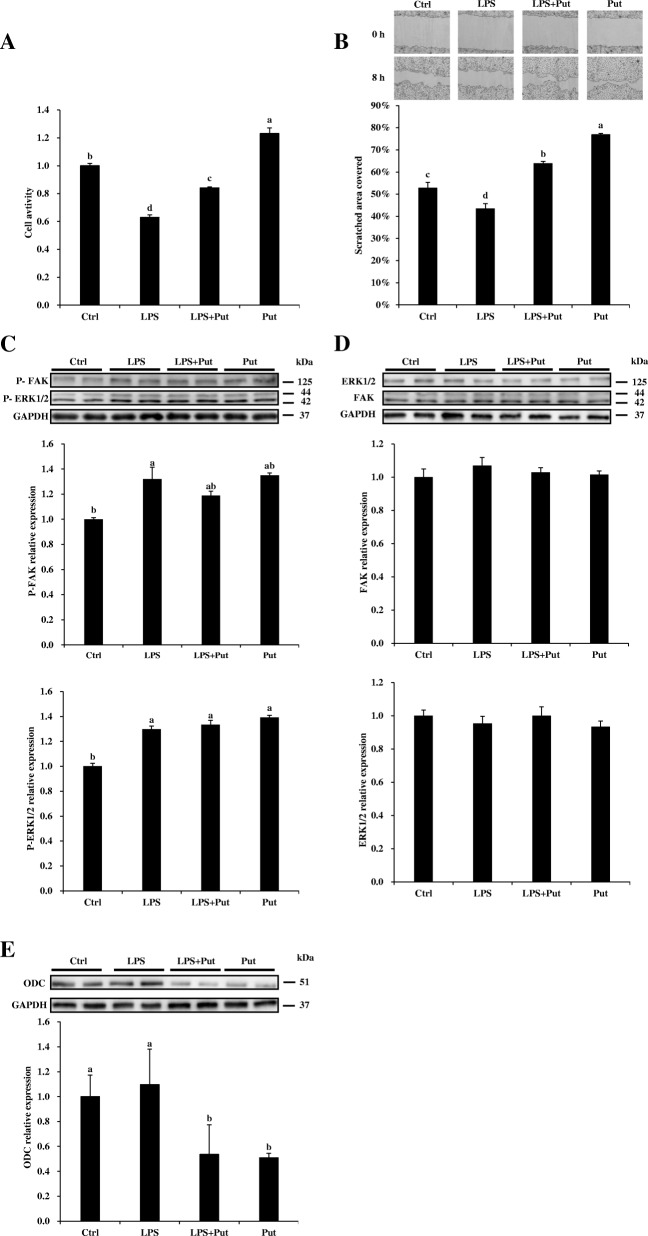
Putrescine rescued the proliferation and migration of LPS-treated IPEC-J2 cells. **a** Cells were seeded in 96-well plates, and pretreated for 48 h with or without 200 μmol/L putrescine. The cells were challenged with or without 100 μg/mL LPS for 4 h, and cell proliferation was measured with the EdU method. **b** IPEC-J2 cells were seeded in 6-well plates, and pretreated for 48 h with or without 200 μmol/L putrescine, followed by the addition of 2 μg/mL mitomycin C for 24 h. The cells were then challenged with or without 100 μg/mL LPS for 4 h before scratching. Images were taken immediately after scratching (0 h) and at 8 h post scratching to calculate area covered by cell migration. The scratched borders were enhanced with black lines. **c** Western blotting of phosphor-ERK1/2 and phospho-FAK. **d** Western blotting of total-Erk1/2 and total-FAK. **e** Western blotting of ODC. Values are means ± SE, *n* = 4. Means with different letters are different (*P* < 0.05). Ctrl, control; IPEC-J2, porcine intestinal epithelial cells; LPS, lipopolysaccharides; P-ERK1/2, phospho-ERK1/2; P-FAK, phospho-FAK; Put, putrescine

The rate of proliferation and migration of IPEC-J2 cells was reduced by LPS challenge. However, this reduction was mitigated by treatment with putrescine (*P* < 0.05) (Fig. 3a, b). LPS challenge enhanced the phosphorylation of ERK1/2 and FAK. Adding putrescine to culture medium had no effect on the phosphorylation of ERK1/2 or FAK in LPS-challenged IPEC-J2 cells (Fig. 3c). LPS challenge or putrescine treatment did not alter the total protein amount of ERK1/2 or FAK (Fig. 3d). The abundance of the ODC protein in cells was not affected by LPS but was reduced (*P* < 0.05) by the addition of putrescine to culture medium (Fig. 3e).

### Effect of dietary supplementing putrescine on expression of proinflammatory genes, and phosphorylation of ERK1/2 and FAK in the jejunal mucosa of weanling piglets

**Fig. 4:**
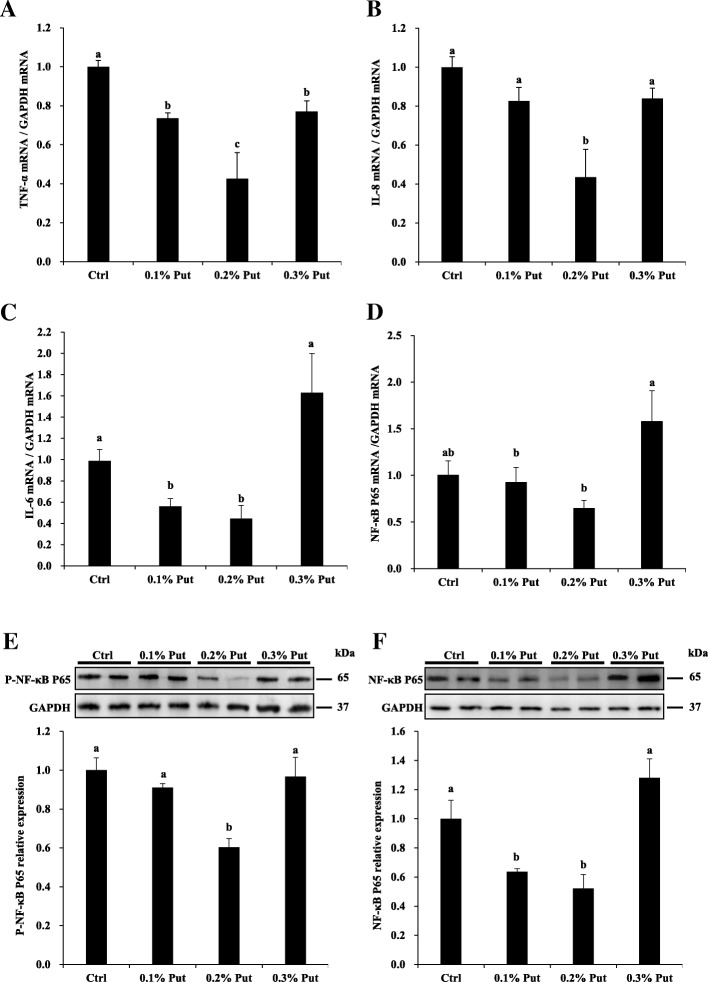
Effects of dietary putrescine supplementation on proinflammatory cytokine expression in the jejunal mucosa of piglets. **a** The mRNA level of TNF-α. **b** The mRNA level of IL-8. **c** The mRNA level of IL-6. **d** The mRNA level of NF-κB p65. **e** The protein level of phospho-NF-κB p65. **f** The protein level of total-NF-κB p65. Values are means ± SE, *n* = 6. Means with different letters are different (*P* < 0.05). Ctrl, control; IL-6, interleukin 6; IL-8, interleukin 8; NF-κB p65: nuclear receptor kappa B p65 subunit; Put, putrescine dihydrochloride; TNF-α, tumor necrosis factor α

Dietary supplementation with 0.1% to 0.3% putrescine dihydrochloride decreased the mRNA levels of an inflammatory cytokine TNF-α in the mucosa of jejunum (*P* < 0.05) (Fig. 4a). Dietary supplementation with 0.2% putrescine dihydrochloride decreased the mRNA levels of another inflammatory cytokine IL-8 in the mucosa of jejunum (*P* < 0.05), but supplementation with 0.1% or 0.3% putrescine dihydrochloride had no effect on IL-8 gene expression (Fig. 4b). Dietary supplementation with 0.1% or 0.2% putrescine dihydrochloride decreased the mRNA levels of inflammatory cytokine IL-6 in the mucosa of jejunum (*P* < 0.05), but supplementation with 0.3% putrescine dihydrochloride had no effect on IL-6 gene expression (Fig. 4c). Dietary supplementation with 0.2% putrescine dihydrochloride tended to reduce the mRNA levels of nuclear receptor kappa B p65 subunit (NF-κB p65) in the mucosa of jejunum (Fig. 4d), and dietary supplementation with 0.1% or 0.2% putrescine dihydrochloride decreased total and phospho-NF-κB p65 protein levels (*P* < 0.05) (Fig. 4e, f).

**Fig. 5:**
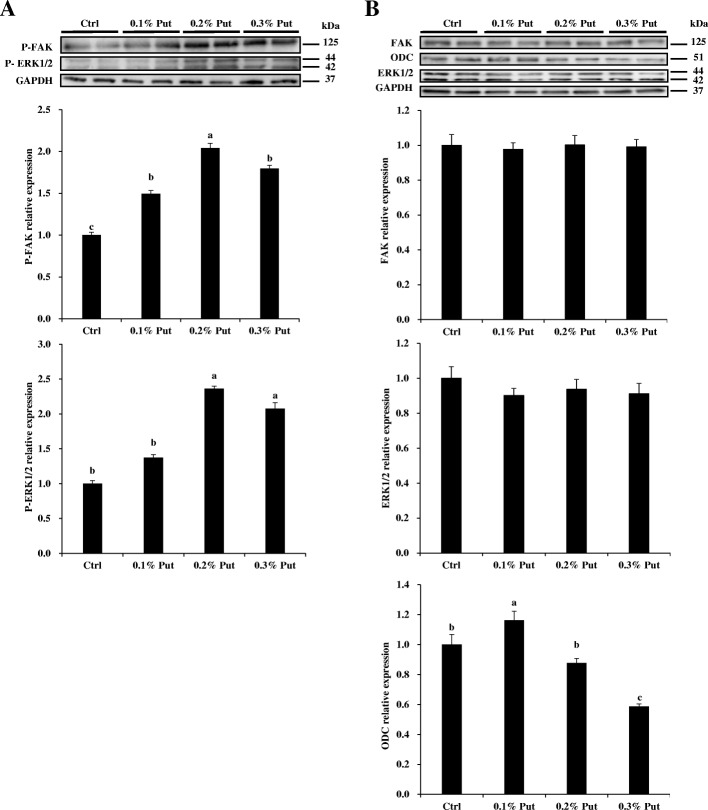
Effects of putrescine supplementation on ERK1/2 and FAK phosphorylation in the jejunal mucosa of piglets. **a** Dietary supplementation with putrescine increased the phosphorylation of ERK1/2 and FAK in the jejunal mucosa of piglets. **b** Dietary supplementation with putrescine had no effect on the total amount of the ERK1/2 and FAK proteins in the jejunal mucosa of piglets, but the mucosal ODC protein abundance was decreased by dietary supplementation with 0.3% putrescine dihydrochloride. Values are means ± SE, *n* = 6. Means with different letters are different (*P* < 0.05). Ctrl, control; ODC, ornithine decarboxylase; P-ERK1/2, phospho-ERK1/2; P-FAK, phospho-FAK; Put, putrescine dihydrochloride

Dietary supplementation with 0.2% or 0.3% dihydrochloride putrescine increased the phosphorylation of ERK1/2 in the mucosa of jejunum (*P* < 0.05) (Fig. 5a). Dietary supplementation with 0.1%, 0.2%, and 0.3% putrescine dihydrochloride all increased the phosphorylation of FAK (*P* < 0.05) (Fig. 5a). However, dietary supplementation with putrescine dihydrochloride did not affect the total protein amount of ERK1/2 and FAK in the mucosa of jejunum (Fig. 5b). The protein level of ODC was decreased by dietary supplementation with 0.3% putrescine dihydrochloride (*P* < 0.05).

## Discussion

Intestinal mucous atrophy, indicated by reduced villous height and deepen crypt, is a key aspect of post-weaning syndrome, and a major cause for intestinal infection and diarrhea in weanling piglets. Here we demonstrated that dietary supplementation with 0.2% putrescine dihydrochloride mitigated intestinal atrophy and decreased the incidence of diarrhea in weanling piglets. Our results were in agreement with other studies on the beneficial effects of polyamines on gut health [7–9, 20]. Importantly, this is the first time to show that exogenous supply of putrescine during the first days of the post-weaning period, a vulnerable time for intestinal development and adaption, alleviated mucosal damage in piglets. However, compared with 0.2% putrescine dihydrochloride, dietary supplementation with 0.3% putrescine dihydrochloride did not confer a beneficial effect on diarrhea incidence and exerted a lesser positive effect on the jejunal morphology. This result may be explained by the negative regulation of ODC expression by putrescine as we observed a lower level of ODC expression with a higher supplemental dose of putrescine dihydrochloride. This negative regulation is mediated by the protein called ornithine decarboxylase antizymer [21]. The mRNA levels of ZO-1 and occludin were increased by supplementation with 0.2% putrescine dihydrochloride, indicating the tight junction of enterocytes was improved by putrescine. This is expected to be beneficial for preventing the envision of pathogens. We provided an effective and practical way to improve gut health for weanling piglets. However, we did not observe improved growth performance by putrescine supplementation for a short time (2 weeks). Longer animal trials (such as 4 or 6 weeks) are warranted to evaluate if the growth performance can be enhanced as the small intestine becomes more mature with dietary supplementation with putrescine.

The molecular mechanisms responsible for polyamine-alleviated mucosal damage remain unclear. Two distinct steps are needed for the repair of damaged mucosa, with the first step being epithelial restitution, which is a cell migration process independent of cell proliferation [22]. During the second phase of repairing, DNA synthesis and cell proliferation are necessary to replace lost cells, which takes a much longer time for healing [23]. Thus, cell migration and proliferation are essential for the repair of mucosal damage. Polyamines are well known for promoting cell proliferation in many cells including intestinal cells [24]. However, little is known about the effect of a polyamine on intestinal cells when they are challenged by an inflammatory condition *in vitro* or *in vivo*. Here we showed that addition of putrescine to culture medium enhanced the proliferation of IPEC-J2 cells, a porcine non-transformed intestinal cell line [16]. Moreover, exogenous putrescine rescued the impaired growth of IPEC-J2 cells caused by DFMO, a specific inhibitor of ODC. This indicates that polyamine is necessary for the growth of IPEC-J2 cells as other cell types. The underlying mechanisms are not fully understood. There is evidence that spermidine acts through post-translational modification to form the hypusine-containing eukaryotic translation initiation factor 5A (eIF-5A), which is essential to cell proliferation [25]. Moreover, putrescine promotes cell proliferation and stimulates the mTOR signaling pathway in porcine trophectoderm cells, a fast dividing cell line as the intestinal epithelial cell line [26]. Here we showed that DFMO inhibited the phosphorylation of ERK1/2; however, this inhibition was eliminated by the addition of putrescine to culture medium. ERK is a key protein in the ERK pathway that transmits signals from growth factors and mitogens to regulate cell growth. Phosphorylation of ERK activates the protein to phosphorylate its downstream proteins such as oncogenes c-fos, c-myc, and c-jun to promote the proliferation of in IEC-6 cells [27]. Our results corroborate the previous study in which polyamine reversed the decreased expression of c-fos, c-myc, and c-jun in DFMO-treated IEC-6 cells [24].

To mimic the intestinal damage *in vivo*, we removed a portion of the cell layer in culture medium by scratching with a pipette tip. We measured the migration rate of cells *in vitro* to mimic the restitution of the damage in the intestinal mucosa. DFMO inhibited the migration of IPEC-J2 cells, but adding exogenous putrescine completely recovered the migration. This demonstrated that putrescine stimulated the restitution of intestinal damage. Moreover, adding exogenous putrescine alleviated the inhibitory effect of DFMO on FAK phosphorylation. FAK is a central mediator of focal adhesion signaling, which couples integrins with multiple cytoskeletal and signaling molecules to play crucial roles in regulating cell migration [28–30]. Here we showed that addition of defactinib (a specific inhibitor of PKA) inhibited the migration of IPEC-J2 cells but the addition of putrescine had no effect, suggesting that PKA is essential to cell migration. It has been demonstrated that polyamines may regulate cell migration through altering K^+^ channel activity, membrane potential, and cytosolic free Ca^2+^ concentration [2]. The TGF-β signaling may also play a role in mediating the effect of polyamines on cell migration, because addition of TGF-β completely restored the ability of IEC-6 cells to migrate after wounding [31].

A novel finding of this study is that putrescine attenuated the inflammatory responses to mitigate intestinal atrophy. Weaning stress is a major factor for initiating inflammatory responses in the small intestine of piglets [10]. To mimic the intestinal inflammation in weanling piglets, we employed a well-established model in which IPEC-J2 cells were challenged with LPS. Here we showed that the expression of proinflammatory cytokine (TNF-α) and chemokine (IL-6, IL-8), which are key mediators in the regulation of immune response, increased dramatically with the stimulation of LPS, indicating the relevance of our cell model. We also demonstrated that adding exogenous putrescine attenuated the expression of TNF-α and IL-8 in response to LPS stimulation. Several lines of evidence suggested that polyamines may be involved in the inflammatory response. Injection of LPS increased ODC activity in the mouse liver [11]; the expression of ODC was upregulated in macrophages following LPS stimulation, but overexpression of ODC in macrophages inhibited the LPS-induced secretion of proinflammatory cytokines [12]; deletion of ODC from myeloids increased gastric and colonic inflammation; however, addition of putrescine reversed macrophage activation [13]. Collectively, these results strongly suggest that polyamines possess an anti-inflammatory function. Here we also showed that LPS decreased cell proliferation and migration. As mentioned above, cell proliferation and migration play essential roles in maintaining intestinal integrity. Excessive inflammation response has a negative effect on the structure and function of intestine in weanling piglets [32]. Importantly, we found that addition of putrescine to culture medium rescued partially the LPS-induced decrease in cell proliferation and migration. Consistent with these *in vitro* results, dietary supplementation with putrescine decreased the mRNA abundances of TNF-α, IL-6, and IL-8 in the jejunum of piglets. Moreover, the protein abundance of NF-κB p65 was reduced by dietary supplementation with putrescine, suggesting putrescine may act through the NF-κB signaling to suppress the inflammatory response in the jejunum of weanling piglets. Nonetheless, our results explain why dietary supplementation with putrescine mitigated the intestinal atrophy in weanling piglets. Considering the similarities in intestinal structure and function between humans and pigs, the results of this work provide a new strategy to ameliorate intestinal dysfunction in weanling piglets and infants.

LPS significantly increased the phosphorylation of ERK1/2 and PKA, but addition of putrescine to culture medium did not alter the LPS-induced increase in the phosphorylation of ERK1/2 and PKA in IPEC-J2 cells. These results suggest that LPS may act through pathways other than ERK or focal adhesion to inhibit cell proliferation or migration. We found that ODC expression was decreased by putrescine, which is consistent with previous studies with other cell types, as a mechanism to regulate intracellular concentrations of polyamines.

It is unknown whether the conversion of putrescine into spermidine or spermine is necessary for putrescine to exert its effects. The concentrations of intracellular spermine increased after the addition of putrescine to culture medium, indicating an active pathway for the conversion of putrescine into spermine in IPEC-J2 cells. There is a suggestion that spermine, but not putrescine, stimulates the proliferation of mammalian cells [33]. Spermine is not as stable as putrescine during storage and is much more expensive than putrescine. Thus, because the pig small intestine can readily form spermidine and spermine from putrescine and methionine [34], putrescine has a distinct advantage over spermine as a dietary supplement for animal feeding.

## Conclusion

Our study showed that mucosal atrophy was ameliorated by dietary supplementation with 0.2% putrescine dihydrochloride in weanling piglets. Putrescine was essential for IPEC-J2 cell growth and migration, and also inhibited the inflammatory response of cells to LPS. These beneficial effects of putrescine were partially mediated by ERK, focal adhesion, and NF-κB signaling pathways. Our results provide a new strategy to improve intestinal integrity and health in weanling piglets and also have important implications for nutritional management of other neonates.

## Additional files

Additional file 1: **Table S1**. The body weight of slaughtered piglets on day 14 of the trial. (DOCX 25 kb)

Additional file 2: **Table S2**. The list of primers used for qPCR. (DOCX 30 kb)

Additional file 3: **Table S3**. The list of antibodies used in western blotting. (DOCX 26 kb)

Additional file 4: **Figure S1**. Effects of putrescine supplementation on tight junction gene expression in the jejunal mucosa of piglets. A. The mRNA level of ZO-1. B. The mRNA level of claudin-1. C. The mRNA level of occludin. Values are means ± SE, *n* = 6. Means with different letters are different (*P* < 0.05). Ctrl, control; Put, putrescine dihydrochloride; ZO-1: zona occludens 1. (PDF 26 kb)

Additional file 5: **Figure S2**. Effect of adding different level putrescine in the medium on the growth of IPEC-J2. The IPEC-J2 cells were seeded in 2% FBS containing DMEM/F12 supplemented with 0, 12.5, 25, 50, 100, or 200 μmol/L putrescine, cell numbers were determined at 0 h, 24 h, 48 h, 72 h, 96 h and 120 h with the CCK-8 kit. Cell growth was presented with plotting diagram (A), and was presented with histogram (B) to show the detail of difference among groups. Growth differences were presented with bar chart. Values are means ± SE, *n* = 4. Means with different letters are different (*P* < 0.05) in the histogram. IPEC-J2, porcine intestinal epithelial cells. (PDF 175 kb)

Additional file 6: **Figure S3**. Mitomycin C suppressed completely cell growth at 24 h of treatment. 0.25 × 10^5^ cell/mL IPEC-J2 cells were seeded in 24-well plates and treated with or without 2 μg/mL mitomycin C for 24 h. Cell number was measured with the CCK-8 kit. Values are means ± SE; *n* = 4. Means with different letters are different (*P* < 0.05). Ctrl, control; IPEC-J2, porcine intestinal epithelial cells. (PDF 93 kb)

Additional file 7 **Figure S4**. ERK inhibitor inhibited IPEC-J2 cells growth and ERK1/2 phosphorylation. A. The IPEC-J2 cells were seeded in 2% FBS containing DMEM/F12 supplemented with or without 200 μmol/L putrescine in the presence or absence of ulixertinib, a specific inhibitor for ERK. Cell numbers were determined at 0 h, 24 h, 48 h, 72 h, 96 h and 120 h with the CCK-8 kit. Ulixertinib inhibited the growth of IPEC-J2 cells from 48 h to 120 h with or without putrescine. B. Western blotting of Phospho-ERK1/2 for different treatment. C. Western blotting of total-ERK1/2. Values are means ± SE, *n* = 4. Means with different letters are different (*P* < 0.05). CCK-8, cell counting kit-8; Ctrl, control; IPEC-J2, porcine intestinal epithelial cells; P-ERK1/2, phospho-ERK1/2; Put, putrescine; Uli, ulixertinib. (PDF 143 kb)

Additional file 8: **Figure S5**. FAK inhibitor inhibited IPEC-J2 cells migration and FAK phosphorylation. A. The IPEC-J2 cells were seeded in 6 well plates, and pretreated for 48 h with or without 200 μmol/L putrescine, followed by the addition of 2 μg/mL Mitomycin C for 24 h. The cells were then treated with or without 100 μg/mL defactinib for 4 h before scratching. Images were taken immediately after scratching (0 h) and at 8 h post scratching to calculate the area covered by cell migration, the scratched borders were enhanced with black lines. B. Western blotting of phospho-FAK**.** C. Western blotting of total-FAK. Values are means ± SE, *n* = 4. Means with different letters are different (*P* < 0.05). Ctrl, control; Def, defactinib; IPEC-J2, porcine intestinal epithelial cells; P-FAK, phospho-FAK; Put, putrescine. (PDF 93 kb)

## Data Availability

All data generated or analyzed during this study are included in this published article [and its Additional files].

## Abbreviations

ADFI: Average daily feed intake
ADG: Average daily gain
CCK-8: Cell counting kit-8
DFMO: Difluoromethyl ornithine
DMEM/F12: Dulbeco’s modified eagle’s medium/F12
ERK1/2: Excellular signal regulated kinase 1/2
FAK: Focal adhesion kinase
G:F: Gain to feed ratio
GAPDH: Glyceraldehyde-3-phosphate dehydrogenase
IL-8: Interleukin 8
IPEC-J2: Porcine intestinal epithelial cells
LPS: Lipopolysaccharides
NF-κB p65: Nuclear receptor kappa B p65 subunit
ODC: Ornithine decarboxylase
TBST: Tris buffered saline with tween 20
TNF-α: Tumor necrosis factor α
V:C: Villus height to crypt depth ratio
