# Supplementary material for: Putrescine mitigates intestinal atrophy through suppressing inflammatory response in weanling piglets
Source: J Anim Sci Biotechnol. 2019 Sep 10;10:69. doi: 10.1186/s40104-019-0379-9 (PMC6734277; doi:10.1186/s40104-019-0379-9)
Supplement: Supplementary file 1 — Table S1. The body weight of slaughtered piglets on day 14 of the trial. (DOCX 25 kb) [file 40104_2019_379_MOESM1_ESM.docx]

**Supplementary Data**

**Supplementary Table** 1 The body weight of slaughtered piglets on day 14 of the trial

| Treatment | Ear tag | Body weight, kg |
| --- | --- | --- |
| Control | 17 | 11.60 |
|  | 24 | 11.30 |
|  | 63 | 10.10 |
|  | 86 | 8.45 |
|  | 91 | 8.80 |
|  | 94 | 8.05 |
| 0.1 % Put | 33 | 13.75 |
|  | 36 | 10.70 |
|  | 23 | 9.05 |
|  | 41 | 8.10 |
|  | 87 | 7.85 |
|  | 96 | 7.45 |
| 0.2 % Put | 21 | 14.15 |
|  | 4 | 12.25 |
|  | 27 | 9.30 |
|  | 64 | 9.10 |
|  | 55 | 8.60 |
|  | 92 | 7.50 |
| 0.3 % Put | 13 | 10.30 |
|  | 34 | 11.65 |
|  | 52 | 9.50 |
|  | 62 | 9.50 |
|  | 77 | 8.30 |
|  | 57 | 7.90 |
