# Supplementary material for: Putrescine mitigates intestinal atrophy through suppressing inflammatory response in weanling piglets
Source: J Anim Sci Biotechnol. 2019 Sep 10;10:69. doi: 10.1186/s40104-019-0379-9 (PMC6734277; doi:10.1186/s40104-019-0379-9)
Supplement: Supplementary file 2 — Table S2. The list of primers used for qPCR. (DOCX 30 kb) [file 40104_2019_379_MOESM2_ESM.docx]

**Supplementary Table 2** The list of primers used for qPCR

| Accession No. | Gene | Primer sequence | Product length, bp |
| --- | --- | --- | --- |
| NM_001244539 | claudin-1 | Forward: 5′-CCTCAATACAGGAGGGAAGC-3′  Reverse: 5′-CTCTCCCCACATTCGAGATGATT-3′ | 76 |
| NM_001206359 | GAPDH | Forward: 5′-GCTTGTCATCAATGGAAAGG-3′  Reverse: 5′-CATACGTAGCACCAGCATCA-3′ | 86 |
| NM_214399 | IL-6 | Forward: 5′-AATGTCGAGGCTGTGCAGATT-3′  Reverse: 5′-TGGTGGCTTTGTCTGGATTCT-3′ | 82 |
| NM_213867 | IL-8 | Forward: 5′-CCGTGTCAACATGACTTCCAA-3′  Reverse: 5′-GCCTCACAGAGAGCTGCAGAA-3′ | 75 |
| NM_001163647 | occludin | Forward: 5′-TCAGGTGCACCCTCCAGATT-3′  Reverse: 5′-TGGACTTTCAAGAGGCCTGG-3′ | 112 |
| NM_001114281 | NF-κB p65 | Forward: 5′-GCGATGAGATCTTCCTGCTG-3′  Reverse: 5′-GCCACTTGTCGGTGCACGTC-3′ | 87 |
| NM_214022 | TNF-α | Forward: 5′-TGGCCCCTTGAGCATCA-3′  Reverse: 5′-CGGGCTTATCTGAGGTTTGAGA-3′ | 68 |
| NM_2340626 | ZO-1 | Forward: 5′-CTGCCAAGTGAAACTGCACA-3′  Reverse: 5′-GACAGAGAACGTGTCAACGC-3′ | 129 |
