# Supplementary material for: Putrescine mitigates intestinal atrophy through suppressing inflammatory response in weanling piglets
Source: J Anim Sci Biotechnol. 2019 Sep 10;10:69. doi: 10.1186/s40104-019-0379-9 (PMC6734277; doi:10.1186/s40104-019-0379-9)
Supplement: Supplementary file 3 — Table S3. The list of antibodies used in western blotting. (DOCX 26 kb) [file 40104_2019_379_MOESM3_ESM.docx]

**Supplementary Table 3** The list of antibodies used in western blotting

| Antibody | Dilution | Company | Cat. # |
| --- | --- | --- | --- |
| Rabbit polyclonal anti-Phospho-FAK (Tyr 397) | 1:1000 | CST* | 3283 |
| Rabbit polyclonal anti-FAK | 1:1000 | CST | 3285 |
| Mouse monoclonal anti-ODC | 1 μg/mL | Abcam | 193338 |
| Rabbit polyclonal anti-Phospho-p44/42 MAPK (P-ERK1/2) (Thr202/Tyr204) | 1:1000 | CST | 9101 |
| Rabbit polyclonal anti-p44/42 MAPK (ERK1/2) | 1:1000 | CST | 9102 |
| Rabbit polyclonal anti- NF-κB p65 | 1:1000 | CST | 9460 |
| Rabbit polyclonal anti-Phospho- NF-κB p65 (Ser536) | 1:1000 | CST | 3033 |
| Rabbit monoclonal anti-GAPDH | 1:1000 | CST | 2118 |
| HRP-linked anti-rabbit IgG | 1:2000 | CST | 7074 |
| HRP-linked anti-mouse IgG | 1:2000 | CST | 7076 |

Note: *CST, Cell Signaling Technology (MA, USA).
