# Supplementary figures and images for: Putrescine mitigates intestinal atrophy through suppressing inflammatory response in weanling piglets
Source: J Anim Sci Biotechnol. 2019 Sep 10;10:69. doi: 10.1186/s40104-019-0379-9 (PMC6734277; doi:10.1186/s40104-019-0379-9)

Supplementary Figure 1

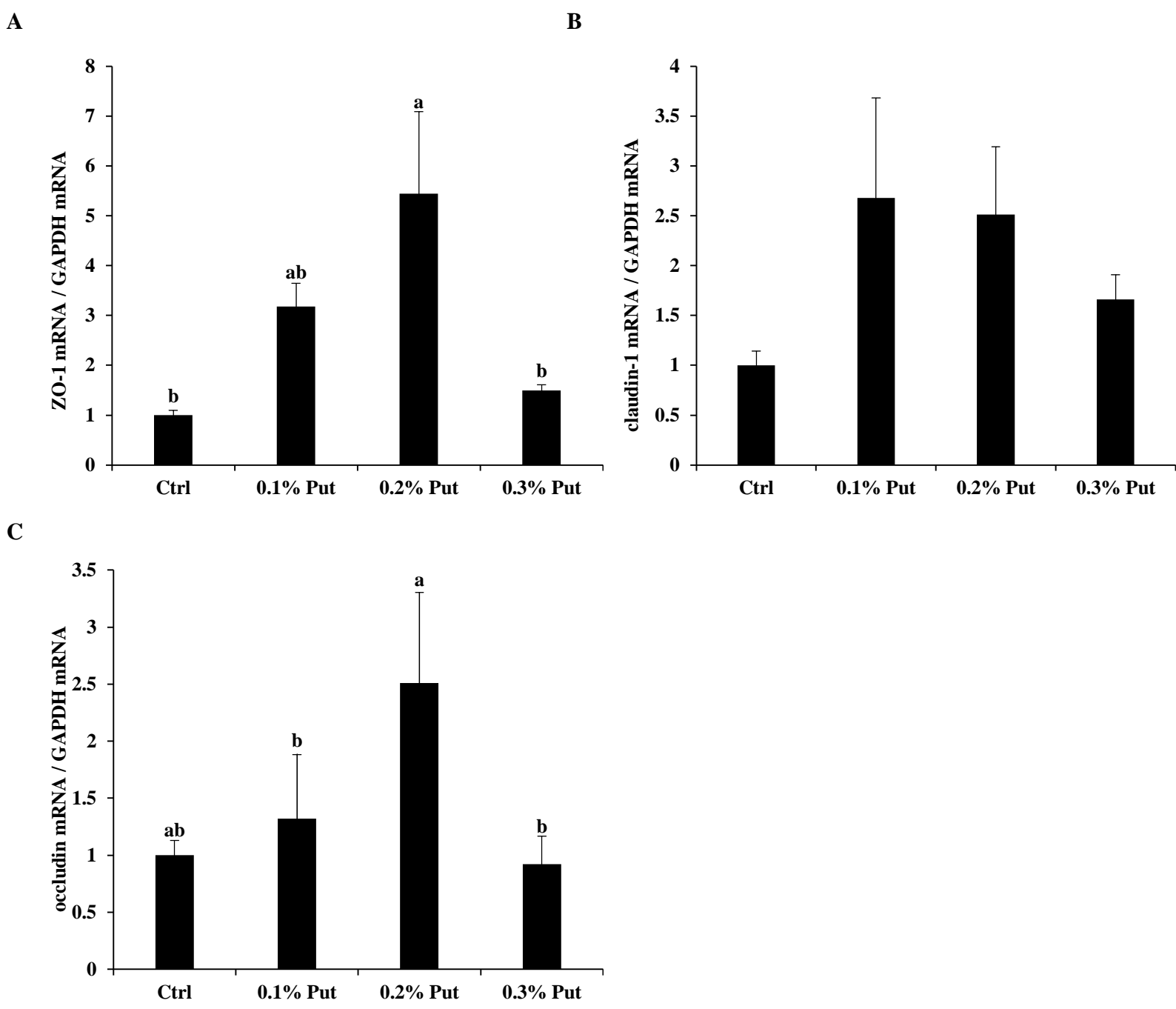

Supplement: Supplementary file 4 — Figure S1. Effects of putrescine supplementation on tight junction gene expression in the jejunal mucosa of piglets. A. The mRNA level of ZO-1. B. The mRNA level of claudin-1. C. The mRNA level of occludin. Values are means ± SE, n = 6. Means with different letters are different (P < 0.05). Ctrl, control; Put, putrescine dihydrochloride; ZO-1: zona occludens 1. (PDF 26 kb) [file 40104_2019_379_MOESM4_ESM.pdf]

Supplementary Figure 2

A

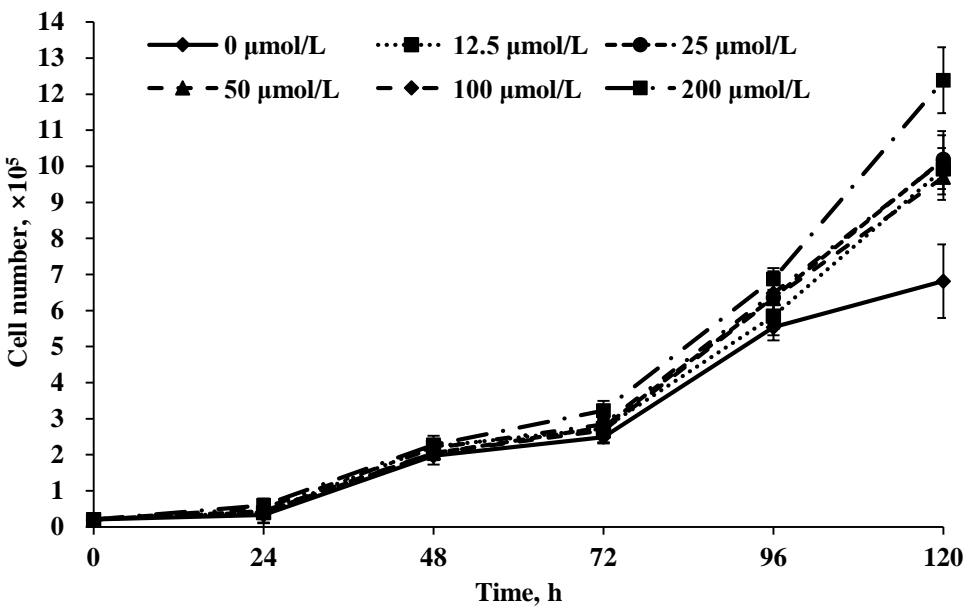

B

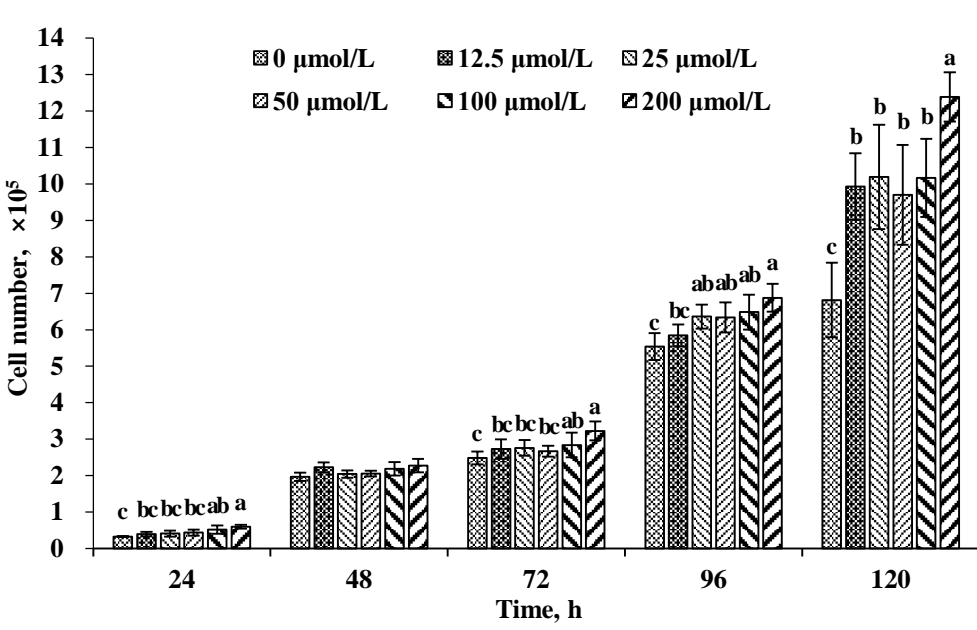

Supplement: Supplementary file 5 — Figure S2. Effect of adding different level putrescine in the medium on the growth of IPEC-J2. The IPEC-J2 cells were seeded in 2% FBS containing DMEM/F12 supplemented with 0, 12.5, 25, 50, 100, or 200 μmol/L putrescine, cell numbers were determined at 0 h, 24 h, 48 h, 72 h, 96 h and 120 h with the CCK-8 kit. Cell growth was presented with plotting diagram (A), and was presented with histogram (B) to show the detail of difference among groups. Growth differences were presented with bar chart. Values are means ± SE, n = 4. Means with different letters are different (P < 0.05) in the histogram. IPEC-J2, porcine intestinal epithelial cells. (PDF 175 kb) [file 40104_2019_379_MOESM5_ESM.pdf]

Supplementary Figure 3

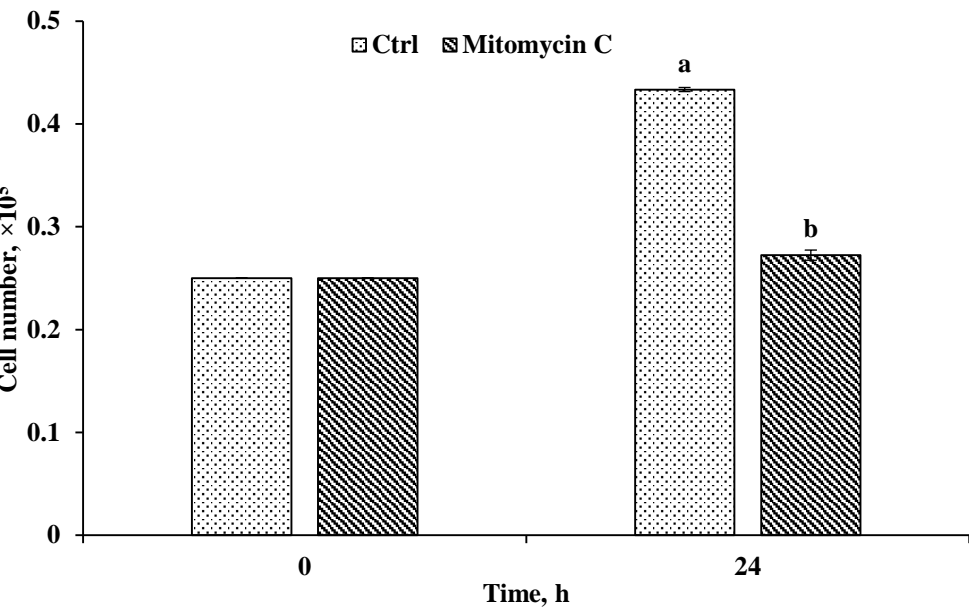

Supplement: Supplementary file 6 — Figure S3. Mitomycin C suppressed completely cell growth at 24 h of treatment. 0.25 × 10^5^ cell/mL IPEC-J2 cells were seeded in 24-well plates and treated with or without 2 μg/mL mitomycin C for 24 h. Cell number was measured with the CCK-8 kit. Values are means ± SE; n = 4. Means with different letters are different (P < 0.05). Ctrl, control; IPEC-J2, porcine intestinal epithelial cells. (PDF 93 kb) [file 40104_2019_379_MOESM6_ESM.pdf]

Supplementary Figure 4

A

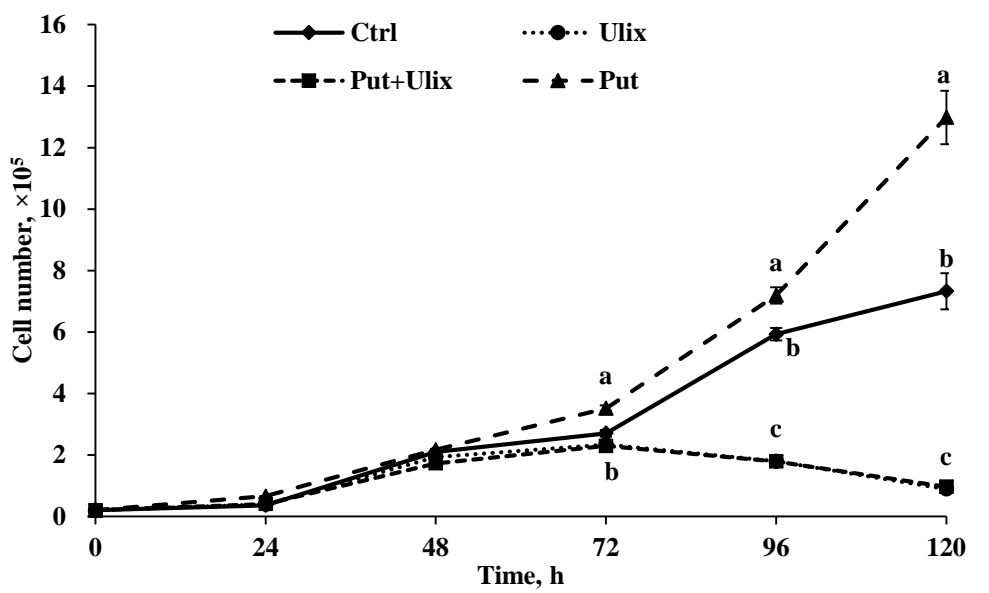

B

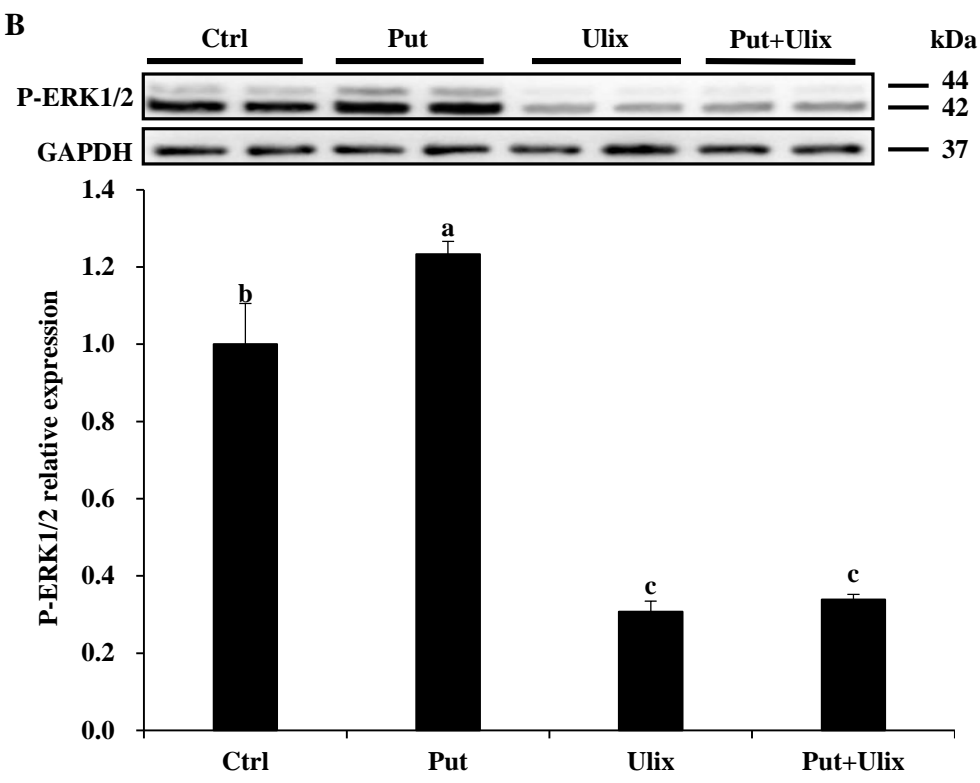

C

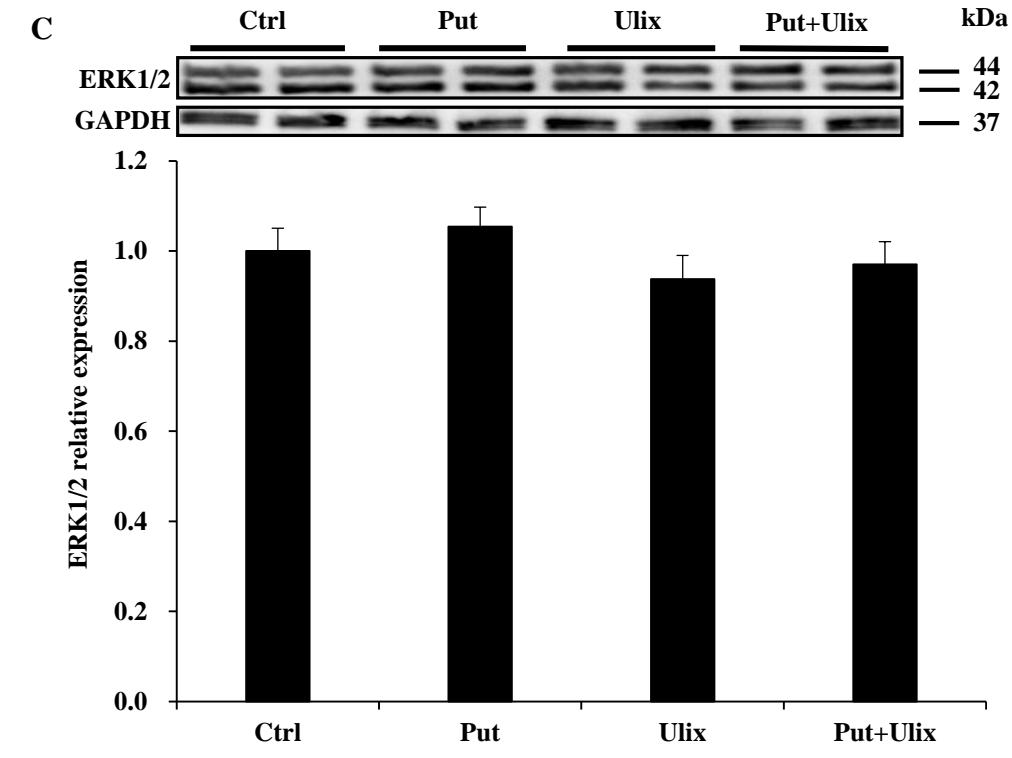

Supplement: Supplementary file 7 — Figure S4. ERK inhibitor inhibited IPEC-J2 cells growth and ERK1/2 phosphorylation. A. The IPEC-J2 cells were seeded in 2% FBS containing DMEM/F12 supplemented with or without 200 μmol/L putrescine in the presence or absence of ulixertinib, a specific inhibitor for ERK. Cell numbers were determined at 0 h, 24 h, 48 h, 72 h, 96 h and 120 h with the CCK-8 kit. Ulixertinib inhibited the growth of IPEC-J2 cells from 48 h to 120 h with or without putrescine. B. Western blotting of Phospho-ERK1/2 for different treatment. C. Western blotting of total-ERK1/2. Values are means ± SE, n = 4. Means with different letters are different (P < 0.05). CCK-8, cell counting kit-8; Ctrl, control; IPEC-J2, porcine intestinal epithelial cells; P-ERK1/2, phospho-ERK1/2; Put, putrescine; Uli, ulixertinib. (PDF 143 kb) [file 40104_2019_379_MOESM7_ESM.pdf]

Supplementary Figure 5

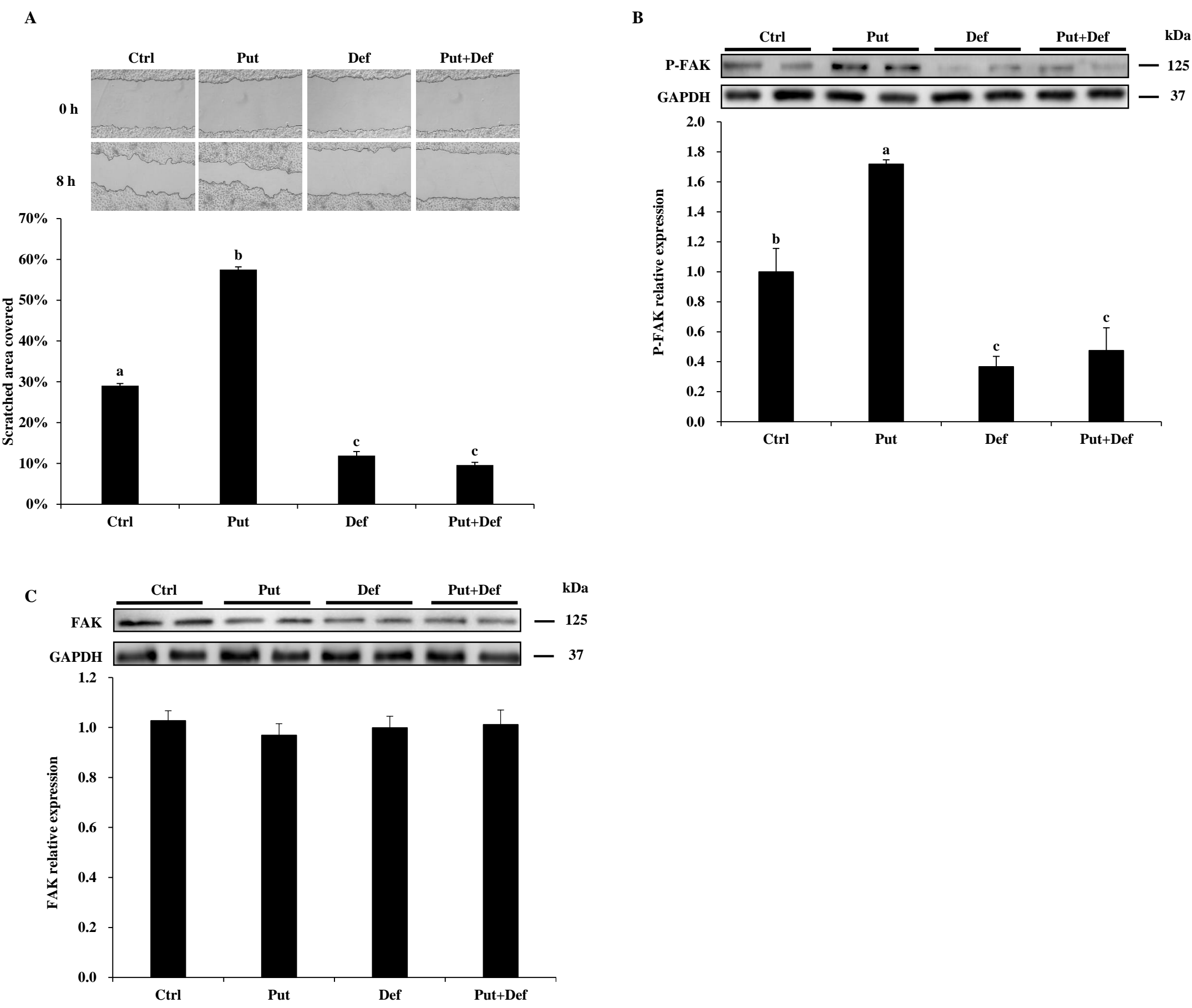

Supplement: Supplementary file 8 — Figure S5. FAK inhibitor inhibited IPEC-J2 cells migration and FAK phosphorylation. A. The IPEC-J2 cells were seeded in 6 well plates, and pretreated for 48 h with or without 200 μmol/L putrescine, followed by the addition of 2 μg/mL Mitomycin C for 24 h. The cells were then treated with or without 100 μg/mL defactinib for 4 h before scratching. Images were taken immediately after scratching (0 h) and at 8 h post scratching to calculate the area covered by cell migration, the scratched borders were enhanced with black lines. B. Western blotting of phospho-FAK. C. Western blotting of total-FAK. Values are means ± SE, n = 4. Means with different letters are different (P < 0.05). Ctrl, control; Def, defactinib; IPEC-J2, porcine intestinal epithelial cells; P-FAK, phospho-FAK; Put, putrescine. (PDF 93 kb) [file 40104_2019_379_MOESM8_ESM.pdf]
